# Primary care delivery of behavioral weight loss services for adults with cardiovascular risk factors: development of pragmatic implementation strategies and results of a randomized feasibility trial

**DOI:** 10.21203/rs.3.rs-2806196/v1

**Published:** 2023-05-15

**Authors:** Ronald T Ackermann, Kenzie Cameron, David Liss, Nancy Dolan, Cassandra Aikman, Amy Carson, Sterling Harris, Kathryn Doyle, Andrew Cooper, Brian Hitsman

**Affiliations:** Northwestern University Feinberg School of Medicine; Northwestern University Feinberg School of Medicine; Northwestern University Feinberg School of Medicine; Northwestern University Feinberg School of Medicine; Northwestern University Feinberg School of Medicine; Fitness Formula Clubs; Northwestern University Feinberg School of Medicine; Northwestern Medicine: Northwestern Memorial HealthCare Corp; Northwestern University Feinberg School of Medicine; Northwestern University Feinberg School of Medicine

**Keywords:** Obesity, Cardiovascular Disease, Diabetes, Primary Prevention, Behavioral Interventions, Implementation, Healthcare Delivery, Primary Care

## Abstract

**Background:**

Intensive lifestyle interventions (ILI) improve weight loss and cardiovascular risk factors, but health systems face challenges implementing them. We engaged stakeholders to cocreate and evaluate feasibility of primary care implementation strategies and of a pragmatic randomization procedure to be used for a future effectiveness trial.

**Methods:**

The study setting was a single, urban primary care office. Patients with BMI ≥ 27 and ≥ 1 cardiovascular risk factor were sent a single electronic health record (EHR) message between December 2019 and January 2020 offering services to support an initial weight loss goal of about “10 pounds in 10 weeks.” All patients who affirmed weight loss interest were pragmatically enrolled in the trial and offered “Basic Lifestyle Services” (BLS), including a scale that transmits weight data to the EHR using cellular networks, a coupon to enroll in lifestyle coaching resources through a partnering fitness organization, and periodic EHR messages encouraging use of these resources. About half (n = 42) of participants were randomized by an automated EHR algorithm to also receive “Customized Lifestyle Services” (CLS), including weekly email messages adapted to individual weight loss progress and telephonic coaching by a nurse for those facing challenges. Interventions and assessments spanned January to July 2020, with interference by the coronavirus pandemic. Weight measures were collected from administrative sources. Qualitative analysis of stakeholder recommendations and patient interviews assessed acceptability, appropriateness, and sustainability of intervention components.

**Results:**

Over 6 weeks, 426 patients were sent the EHR invitation message and 80 (18.8%) affirmed interest in the weight loss goal and were included for analysis. EHR data were available to ascertain a 6-month weight value for 77 (96%) patients. Overall, 62% of participants lost weight; 15.0% exhibited weight loss ≥ 5%, with no statistically significant difference between CLS or BLS arms (p = 0.85). CLS assignment increased participation in daily self-weighing (43% versus 21% of patients through 12 weeks) and enrollment in referral-based lifestyle support resources (52% versus 37%).

**Conclusions:**

This preliminary study demonstrates feasibility of implementation strategies for primary care offices to offer and coordinate ILI core components, as well as a pragmatic randomization procedure for use in a future randomized comparative trial.

## Introduction

Moderate-intensity physical activity and just 10 to 15 pounds of weight loss improves blood glucose, blood pressure, and cholesterol and reduces the need for medications to control those cardiovascular risk factors.^1-6^ Achieving and maintaining weight loss is difficult for most people, particularly if the environment surrounding them is not supportive of healthy eating and physical activity.^7^ Adults are more likely to achieve weight loss and activity goals when supported by behavioral interventions involving recurring contact with a behavioral coach over months to years.^1,2^ Such “intensive lifestyle interventions” (ILI) improve health related quality of life, reduce work absence, and lower future healthcare utilization and costs.^8,9^

U.S. health systems are a natural partner for ILI implementation because 5 in 6 adults complete office visits each year,^10^ during which cardiovascular risk factors such as body weight, blood pressure, glucose, and cholesterol are routinely assessed. Health systems also have incentives to intervene before cardiovascular risk factors cause symptoms, functional impairments, or costly health complications.^9^ However, health systems face challenges implementing ILI,^11,12^ which require dedicated personnel, space, supplies, and technologies that generally do not “fit” within the existing structure and routines of healthcare delivery.

Past research has investigated strategies to simplify the role of health systems in ILI delivery. Examples include reducing the frequency or duration of behavioral coaching contacts or shifting the source of coaching to outside healthcare settings, such as via wellness professionals employed by community organizations or virtual coaching delivered using a computer or smartphone.^13-17^ These strategies have slightly lower effectiveness than more intensive interventions studied in randomized controlled trials (RCTs),^18^ but also cost less and reach more people.^8,19,20^ Despite increasing numbers of ILI programs nationally, clinicians refer very few patients to ILI, and most people who may benefit remain unaware.^19^ There is an immense need for research that identifies feasible and acceptable strategies for health systems to implement ILI.

One approach used to improve implementation of multi-component behavioral interventions is to distill them to their core components, and devise simpler implementation strategies for each component that both fit within health system constraints and offer a broader array of ways for patients to engage.^21,22^ Such an approach may expand reach to patients who are initially unable or unwilling to enroll when referred to a full-scale program offered in a single format or location. Core components of ILI include support for: an initial *weight loss goal* of at least 5 to 10 kg (11 to 22 pounds) at a pace of about 0.5 to 2 pounds per week;^1^ resources and training for individuals to *self-monitor* dietary, activity, and weight changes;^23^ and *longitudinal coaching* (typically with contact every 1 to 4 weeks). Coaching serves to create accountability,^24^ develop self-regulation and problem-solving skills,^25,26^ suggest refinements to behavioral strategies, and build self-efficacy and mastery over behaviors;^23^ coaching can be effective when offered face-to-face or via a technology platform, with individual participants or groups.^1^

Over the past 3 years, we engaged with health system and fitness industry leaders, managers, and service professionals to co-create and evaluate the feasibility of an array of practical implementation strategies for health systems to support ILI core components. To our knowledge this is the first such effort in which all intervention components are imbedded within or coordinated directly by an Electronic Health Record management system. We report on the collaborative approach used for creation of the ILI implementation strategies, as well as preliminary data for feasibility and acceptability of this approach.

## Methods

Our research involves both stakeholder-engaged “co-creation” (i.e., development and refinement) of implementation strategies using collaborative design principles and a small pilot trial to demonstrate feasibility and acceptability of the strategies in a single general internal medicine practice setting in downtown Chicago, Illinois. The trial involved pragmatic recruitment, enrollment, and data collection strategies. Eligible patients were enrolled from December 2019 – January 2020; data were collected through January 2021. The experimental services were offered to patients beginning January 2020, and access to some services (details below) were interrupted by the coronavirus pandemic stay-at-home order in March 2020.

### Stakeholder Engagement

From September 2016 to December 2019, we engaged stakeholders to consider research evidence for ILI core components^1,2,23^ and co-create implementation strategies for sustainable delivery of each core component in busy primary care practice settings. Recurring meetings of a “design team” included behavioral and social scientists, health system nursing supervisors, a practicing primary care physician, two wellness program leaders from the partnering fitness club network, and health system employees who develop and manage new tools and applications within the electronic health record (EHR) system. Individual meetings were held with additional stakeholders, including health system leaders, medical group and practice leaders, and the CEO and wellness program leaders from a regional fitness club network. Research staff members organized meetings, kept detailed notes, verified recommendations and action steps with participants, and integrated ideas into an array of design themes and concepts that informed intervention prototypes, pretests, and refinements.^11^

### Feasibility Trial Participants and Recruitment

A clinical data manager utilized EHR reporting tools to generate lists of eligible patients. Inclusion criteria were: age 18–75 years; ≥1 log-in within the past 6 months to MyChart (i.e., a secure EHR access portal for patients to view their chart and exchange information with care team members); completion of ≥ 1 office visit in the past 6 months at the participating primary care practice site; measured weight and height at the most recent office visit indicating body mass index (BMI) ≥ 27.0 kg/m2; and ≥ 1 cardiometabolic risk factor (high blood pressure; abnormal blood cholesterol, prediabetes, or type 2 diabetes). Patients were ineligible if their last blood pressure was ≥ 180/105 mmHg or last hemoglobin A1c was ≥ 10.9%. Patients were also ineligible if they received cancer treatment in the past 2 years, were hospitalized in the past 3 months, were pregnant, or had personal history of an eating disorder or serious mental illness.

The data manager used EHR messaging tools to send each physician a list of their own eligible patients, asking them to reply within 1 week if they wished to remove patients whom they believed offering support for lifestyle change may be inappropriate. After 1 week, the data manager used the EHR's batch patient messaging tool to transmit a standardized outreach “goal setting” message to each patient remaining on the list. The message encouraged healthy eating and physical activity changes with a weight loss goal, and it offered free-of-charge access to technologies and coaching support to help recipients achieve the goal. The message also prompted patients to click an embedded link if they wished to adopt a weight loss goal and receive support. Because limited resources were available to support intervention costs during feasibility testing, we limited recruitment by releasing MyChart messages to 100–200 patients every 2 weeks until a target sample size of 80 patients had affirmed interest in receiving the intervention services. All 80 patients were included in the pragmatic trial evaluation; all received a bundle of Basic Lifestyle Support (BLS) services (below), and about half (N = 42) of patients were randomly assigned by a “silent” EHR algorithm to also receive additional, more Customized Lifestyle Support (CLS) services (below).

### Implementation Strategies

Design goals were to co-create ILI implementation strategies that are evidence-based,^1,2^ and perceived by stakeholders as technically feasible, acceptable, and financial sustainable.^27^ This approach followed collaborative design principles,^11^ and produced ILI implementation strategies for support of weight loss goal setting, self-weighing, and recurring coaching support. Stakeholders advised using the EHR to identify eligible patients, to message them to encourage weight loss goal setting, inspire accountability, and increase engagement in support services, as well as to track and monitor progress as means to offer alternative, higher intensity services to patients facing challenges with automated support alone (**Additional File Table 1**). Stakeholders cautioned that encouragement of a weight loss goal in a MyChart message could be perceived as inappropriate by some patients; they advised framing the goal and offering resources as means to improve cardiovascular risk factor control. They also advised recommending a relatively simple initial goal of “about 10 pounds over 10 weeks,” believing this would be acceptable, appropriate, and achievable for most patients. Stakeholders advised exploring the feasibility of receiving data from a wireless scale and analyzing that data directly within the EHR as means to inform more personalized feedback about self-weighing, as well as to increase accountability and to identify and target patients needing alternative forms of intervention support. They advised providing all patients with free-of-charge access to coaching services offered by a partnering fitness organization and assigning a practice nurse to provide telephonic coaching to patients who do not make progress towards their weight goal. They emphasized that we evaluate the quality of all new resources, if patients perceive them as appropriate, and if their costs are sustainable.

The resulting intervention components (**Additional File 1**) were all deployed or coordinated by the EHR. Interventions commenced when a patient received the introductory MyChart goal-setting message (above) and clicked the link to affirm interest in a weight loss goal. All 80 patients received a bundle of Basic Lifestyle Support (BLS) services. Specifically, they were shipped a wireless weight scale (eScale), which had been programmed to transmit weight data back to their electronic chart using cellular networks. Each patient received a follow-up MyChart message, with simple instructions for using the scale to weigh themselves daily, as well as a “coupon code” to enroll free-of-charge in longitudinal lifestyle coaching and support resources offered by a regional fitness organization partner. Patients could choose to access the referral services in 2 formats: face-to-face access to a dietician and fitness coach at 11 facility locations or fully “virtual” access to the same professionals via video chat with a smartphone, tablet, or personal computer. The face-to-face option provided full health club facility access, and the virtual option provided a suite of healthy food preparation and physical activity tips, tracking tools, and videos. Each patient also received instructions for messaging a dedicated nurse-coach at the primary care practice if they had problems using the support services or if they wanted more personalized support for reaching dietary, physical activity, or weight loss goals.

Anticipating that many patients may benefit from more frequent nudging and greater personalization of the behavioral support, the design team recommended two additional intervention components with these goals. Because more intensive services introduce additional implementation challenges and cost, we evaluated these components by offering them only to about half (n = 42) of patients who had been randomized to receive “Customized Lifestyle Support” (CLS) services. Specifically, for the first 12 weeks, each patient in the CLS arm was sent an automated weekly MyChart message with coaching tips customized to their use of the eScale in the prior week, whether they had made weight loss progress (defined as ≥ 0.5 pounds of weight loss from the first to last weight measure during the prior week), and whether they had enrolled in referral-based services offered by the fitness partner. A practice nurse used an EHR dashboard to monitor the entire CLS group and attempted to call patients to offer customized “step-up” telephone coaching if either: (1) they had not used their scale in the prior week; or (2) for 2 consecutive weeks they had not achieved ≥ 0.5 pounds of weight loss.

### Data Collection

We collected quantitative data from administrative sources and qualitative information from interviews with randomly selected subsets of representative patients. Beginning approximately 12 weeks after launch of the trial, we conducted semi-structured telephone interviews with 15 representative patients to assess perceptions of the appropriateness, ease of access, ease of use, acceptability, desirability, credibility, and usefulness of the intervention components in helping them achieve health related goals.^11,28^

Several electronic sources provided data for patient characteristics and outcomes. EHR data spanning +/− 15 months of the launch of the trial provided information on the baseline characteristics, service utilization patterns, and metabolic and biometric outcomes. The partnering fitness network provided data about patient participation in the two coaching support options and facility use. Analysis of body weight changes utilized data from the EHR and electronic scales (eScales). The last EHR weight measure prior to each patient’s randomization date served as the baseline body weight, and the EHR weight measure closest to the date of randomization plus 6 ± 3 months served as the follow-up weight. For patients who did not have such a follow up EHR weight value, we estimated the 6-month value as one-half the difference between the baseline value and the EHR weight value closest to 12 ± 3 months. For participants missing both a 6- and 12-month weight value in the EHR, we used the eScale weight value closest to 6 ± 3 months. Finally, if individuals were missing all those weight values, we assumed that the participant failed to reach 5% weight loss.

Multiple data sources were used to describe intervention activities and activity-related costs. Input from health system leaders and study team members identified the specific personnel, supplies, contract services, and facility and administrative inputs needed to implement each intervention component. Administrative data systems captured the number and type of contacts made between nurses and study participants. Staff members were interviewed to estimate the amount of time they spent supporting different intervention activities over 6 and 12 months. Costs of eScales, community intervention program fees, and development costs for health IT components were determined from invoice amounts. Maintenance costs for IT support was estimated from interviews of IT staff members, who were asked to estimate hours per week spent supporting the intervention components.

### Analysis

We examined univariate and bivariate descriptive statistics and conducted chi-square tests to compare all patients randomized to CLS and BLS arms on the proportion that reached the dichotomous primary outcome of ≥ 5% weight loss at 6 months. Outcome assessors were blind to the randomized study group assignment at the time of analysis. Transcripts of interviews with patients were analyzed to summarize perspectives raised about acceptability, appropriateness, and feasibility of ILI implementation strategies. Example themes and representative quotes were selected by group deliberation and consensus.

## Results

### Feasibility and Reach of Trial Recruitment

**Figure 1:**
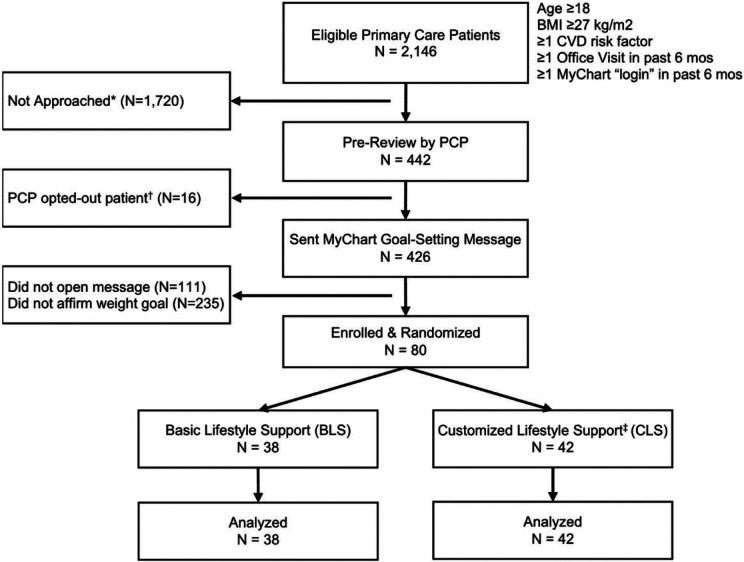
Feasibility Trial Flow BMI: Body Mass Index; CVD: Cardiovascular Disease; MyChart is the patient access portal for electronic chart access and secure messaging with clinicians and staff of the healthcare system; PCP: Primary Care Provider; * Batches of 100-200 sequential patients from the eligibility list were sent a single MyChart message offering support for weight loss; this procedure was repeated every 2 weeks until 80 patients (the targeted sample size) had accepted the services; patients remaining on the list were not approached; † Prior to sending a MyChart message to eligible patients, the data manager sent names to their primary care clinician for pre-review; 16 (3.6%) were not approached at their physician’s request; ‡ Eligible patients who were pragmatically randomized to receive additional “Customized Lifestyle Support” services also received weekly email messages adapted to individual weight loss progress and telephonic coaching by a nurse for those facing challenges (see text for details)

The eligibility list for the feasibility trial included 2,146 patients. Over 6 weeks, the clinical data manager sent names of 442 patients to 34 primary care clinicians for pre-review; clinicians asked that only 16 (3.6%) of these patients not be contacted. A single outreach Mychart message was sent to each of the remaining 426 patients, of whom 315 (73.9%) opened the message within 4 weeks, and 80 (18.8%) affirmed interest in the weight loss goal (Fig. 1).

Demographic characteristics of the 80 participants (63% women; 29% non-Hispanic Black; 14% Hispanic/Latinx; 30% age ≥ 65) approximated the characteristics of the overall target population, with slight over-representation of women and those identifying as Hispanic/Latinx, non-Hispanic Black, or non-Hispanic Asian (Table 1). In the setting of relatively small sample sizes, trial patients randomized to CLS were more often than BLS patients to be older than 45, male, and Hispanic/Latinx. CLS patients were also more likely to have prediabetes and a higher Charlson comorbidity score.

### Engagement in Intervention Components

All but 3 (3.8%) patients were able to initialize their eScale and register a baseline weight. Over the 12-week MyChart messaging phase of the intervention, 11 (14%) of participants did not use their scales for self-weighing, 7 (9%) participated in weighing in some but not all weeks, and 62 (78%) engaged in self-weighing during all 12 weeks. Self-weighing daily (5 to 7 days per week) was performed by 26 (33%) of patients during all 12 weeks; more patients assigned to CLS (customized weekly messaging) performed daily self-weighing in all weeks (43% versus 21% of those offered BLS).

Overall, 36 (45%) patients (37% of the BLS arm and 52% of the CLS arm) enrolled in one of the two referral-based coaching platforms offered by the fitness industry partner; 22 (27.5%) elected the face-to-face option and 14 (17.5%) elected fully virtual access. Patients enrolled in these services between 15 and 77 days after receiving the initial outreach MyChart message. Access to fitness facilities (i.e., the face-to-face delivery option) was disrupted by a coronavirus pandemic stay-at-home order beginning the third week of March 2020. At that time, patients had been enrolled in the referral-based services for 19 to 75 days; the 22 patients in face-to-face support had completed an average of 3 (range 1 to 6) individual coaching sessions and attended a partnering fitness facility 0 to 24 times; the 14 patients receiving “virtual” support had completed an average of 4 (range 2 to 5) individual coaching sessions prior to the pandemic but were able to complete an average of 3 additional (range 0 to 5) coaching sessions after the pandemic began.

Among patients randomized to CLS, 41 (98%) received at least one step-up telephonic nurse coaching call; 19 (45%) received ≥ 5 nurse calls (maximum 11 calls received) over the 12-week intensive support phase.

### Weight loss feasibility

Despite lower than usual office visit rates during the early phase of the coronavirus pandemic, 44 (55%) of feasibility trial patients had an office weight measure recorded in the EHR at 6 ± 3 months from their trial enrollment. Among the remaining 36 patients, 26 (72%) had a weight recorded at 12 ± 3 months, and of the remaining 10 patients, 7 (70%) had an eScale weight received at 6 ± 3 months. Thus, our pragmatic data capture strategy enabled estimation of a 6-month weight change for 77 (96%) of all trial participants. The average time between trial enrollment and the weight used for 6-month data analysis was 184.5 days. Overall, 62% of participants had lower weight at follow up than at baseline; 15.0% exhibited a weight loss of ≥ 5% of baseline weight, with no statistically significant difference between CLS or BLS arms (p = 0.85).

Using eScale data and assuming that the 14% of patients who did not self-weigh also did not lose weight, 28 (35%) of all participants achieved ≥ 10 lbs of weight loss (i.e., the initial goal recommended by the MyChart message) and 24 (30%) achieved ≥ 5% of weight loss at *some* point during the 12-week automated messaging phase. Thus, half of patients who achieved a 5% weight loss during the 12 weeks of active support maintained the loss at 6 months.

### Intervention Delivery Costs

Direct medical costs associated with offering each intervention component are summarized in **Additional File Table 3**. Mean health system costs to offer the intervention as delivered during the feasibility trial were estimated at $335 per person over the first 6 months ($284 for BLS patients; $382 for CLS patients) and $150 per person over the second 6 months ($124 for BLS; $174 for CLS patients).

### Patient Perceptions about Acceptability of Practice Components

Among the 80 feasibility trial participants, 30 were randomly selected and approached before 15 agreed to complete the telephone interview; 11 were unreachable, 5 declined participation, 2 asked to be recontacted at a future time. Respondents included 5 men and 10 women, with demographic and clinical characteristics generally representative of the overall target population (Table 1). Patient perceptions are summarized below; representative quotes and design implications are organized in **Additional File Table 2**.

Most patients described the Mychart messages and website resources as generally easy to understand and navigate. However, some reported feeling frustrated or less engaged by not having a clear understanding upfront for how the different intervention components were coordinated or should be utilized. **Trust in the person or organization providing lifestyle support services** emerged as an important theme. Importantly, some patients expressed being distrustful when offered resources for “free” from a commercial partner, unless it was clear that their doctor or the health system were recommending and coordinating the activities. Others indicated that they had not initially realized they could access dietary and physical activity coaching at the partnering fitness organization and were unsure how to engage with the practice nurse if they had problems.

Nearly all patients reported that an initial weight loss goal of about 10 pounds in 10 weeks was appropriate and achievable. Some expressed interest in larger weight loss goals and access to services beyond 10 to 12 weeks. Many patients reported that their **weight loss goal was motivated by other health or social goals**, such as reducing the need for more pills to treat high blood pressure.

Several patients highlighted the **critical importance of supportive accountability** in successfully changing behavior. They emphasized the practice nurse, registered dietitian, and physical trainers as key sources of supportive accountability, more so than the automated messages. Patients expressed **strengths and limitations of the automated messages**, highlighting that the messages were a helpful reminder or source of encouragement when making progress but were insufficient or even demoralizing when not making progress.

Many patients viewed self-weighing as an additional source of accountability. Some expressed interest in more elaborate electronic scales or integration with other technologies, such as smartphone apps that provide immediate and customized support. Other patients indicated that they preferred support coming from a member of the healthcare or fitness center team. Generally, patients expressed **frustration when they viewed eScales (and self-weighing) as a direct source of support**, rather than as a means for healthcare providers and coaches to track and customize their support. Importantly, some viewed daily weighing as a source of stress and felt that no matter how closely they followed diet and physical activity plans, their daily weight remained unpredictable. Several patients highlighted failure expectations stemming from negative experiences with prior weight loss attempts. Those patients recommended a greater **focus on recognizing and addressing failure expectations**, as well as de-emphasizing a focus on the daily weight readings as a sign of success or failure, while offering more immediate support beyond automated messaging. Relatedly, some patients highlighted how emotions have a larger influence on dietary behaviors than reminders to eat healthy. Those patients recommended greater **focus on acknowledging and addressing emotions both as drivers and consequences of attempts to lose weight** and change diet.

Finally, all patients indicated that the abrupt start of **the coronavirus pandemic presented a profound barrier to healthy behaviors and weight loss**. Many indicated frustration with becoming more sedentary, adopting ‘stress eating’ behaviors, and gaining weight. The pandemic restrictions eliminated access to fitness facilities, which added to frustration.

## Discussion

This preliminary work demonstrates the feasibility of several pragmatic implementation strategies for primary care offices to support the core components of intensive lifestyle interventions. After a single outbound MyChart message was sent to encourage a weight loss goal by overweight or obese adults with cardiovascular risk factors, 3 in 4 recipients opened the message, and 25% of those who opened it affirmed interest in receiving support services lose weight. Among those patients, 30% achieved ≥ 5% weight loss in the first 12 weeks and, despite the disruptive impacts of a pandemic, about 15% still exhibited ≥ 5% weight loss at 6 months, at a time when weight *gain* was common.^30-32^

Weekly “nudging” of patients with a customized MyChart message plus step-up telephone coaching by a practice nurse increased participation in daily self-weighing (43% versus 21% of patients through 12 weeks) and enrollment in referral-based lifestyle support resources offered by a partnering fitness organization (52% versus 37%). Though this small feasibility trial did not demonstrate whether patients are more likely to reach weight loss goals when provided these more customized services, this approach warrants further study.

Our work is consistent with other recent studies of pragmatic, technology-facilitated lifestyle interventions in primary care settings,^13-17^ but we are unaware of other examples where the intervention components have been fully embedded within an EHR system. Our approach employed design thinking with service professionals, health system leaders, health payers, and researchers to create practice components that not only are evidence-based but also desirable to implementers, technically feasible, and financially sustainable.^11^ Design prototypes were iteratively pre-tested and refined with stakeholder collaboration, and a small feasibility trial demonstrated strong promise for both effectiveness and sustainability.

Our work also builds on past research evaluating referral linkages between health systems and community-delivered intensive lifestyle interventions.^33-36^ However, most past research focused on referrals to a comprehensive program bundle, such as the National Diabetes Prevention Program (NDPP), often offered in a single format or location.^18,37^ Offering a multi-component program like NDPP requires considerable community organizational capacity and financial investment, which imposes implementation barriers,^38,39^ particularly when enrollment is low or third party payment is limited. Another unique aspect of our approach was to distill ILI programs down to core components and to develop implementation strategies for each component to minimize demands placed on the implementing organization.^22^ We also considered howto offer the resulting services using an array of different channels (e.g., MyChart, telephone, smartphone application, and fitness facility) to maximize reach by appealing to different segments of the target population.

Conceptually, the development of discreet implementation strategies for individual ILI components has appeal if they successfully minimize workflow disruptions, distribute costs of the new activities across different partners, and map costs to existing pathways for health payer reimbursement. Possible disadvantages are that the individual components may not prove as effective in isolation, and additional strategies must be developed to coordinate staff roles, technologies, and patient transitions among the practice components. Some patients may still face access barriers if, for example, referral-based resources are limited or health payers do not provide reimbursement for remote weight monitoring or lifestyle counseling activities by non-physicians.^40,41^ It will be important for future research to evaluate these dimensions of implementation in the context of a larger and more definitive effectiveness trial.

This preliminary research indicates that strategies to implement ILI core components are feasible for primary care practices and generally acceptable to patients. Further research should seek to optimize implementation strategies to maximize feasibility, acceptability, sustainability, and reach, as well as to demonstrate effectiveness and cost-effectiveness compared with alternative service models. Engaging stakeholders in co-creation of these strategies holds promise to improve both implementation and reach of ILI components in new ways that achieve population health and health equity for millions of Americans who are currently overweight or obese and already engaged by the healthcare sector.

## Figures and Tables

**Table 1.** Baseline Characteristics of Study Patients

Baseline Characteristics*	Eligible, Approached (N = 426)	Affirmed Weight Goal, Enrolled (N = 80)	Basic Lifestyle Support (BLS) (N = 38)	Customized Lifestyle Support (CLS) (N = 42)	Completed Interview^†^ (N = 15)
**Age Group, N (%)**					
Age 18-44	61 (14.3%)	12 (15.0%)	8 (21.1%)	4 (9.5%)	0 (0.0%)
Age 45-64	230 (54.0%)	44 (55.0%)	18 (47.4%)	26 (61.9%)	12 (80.0%)
Age 65+	135 (31.7%)	24 (30.0%)	12 (31.6%)	12 (28.6%)	3 (20.0%)

**Gender, N (%)**					
Female	234 (54.9%)	50 (62.5%)	26 (68.4%)	24 (57.1%)	10 (66.7%)
Male	192 (45.1%)	30 (37.5%)	12 (31.6%)	18 (42.9%)	5 (32.3%)

**Race/Ethnicity, N (%)**					
Hispanic/Latinx	44 (10.3%)	11 (13.8%)	3 (7.9%)	8 (19.1%)	4 (26.7%)
Non-Hispanic Black	113 (26.5%)	23 (28.8%)	12 (31.6%)	11 (26.2%)	6 (40.0%)
Non-Hispanic Asian	14 (3.3%)	6 (7.5%)	2 (5.3%)	4 (9.5%)	0 (0.0%)
Non-Hispanic White	217 (50.9%)	34 (42.5%)	16 (42.1%)	18 (42.9%)	4 (26.7%)
Other	38 (8.9%)	6 (7.5%)	5 (13.2%)	1 (2.4%)	1 (6.7%)

**Qualifying Diagnoses, N (%)**					
Prediabetes	128 (30.1%)	30 (37.5%)	19 (50.0%)	11 (26.2%)	6 (40.0%)
Diabetes	138 (32.4%)	19 (23.8%)	6 (15.8%)	13 (31.0%)	5 (33.3%)
High Blood Pressure	324 (76.1%)	62 (77.5%)	29 (76.3%)	33 (78.6%)	13 (86.7%)
Dyslipidemia	341 (80.1%)	61 (76.3%)	27 (71.1%)	34 (81.0%)	11 (73.3%)

Charlson Score^‡^,	1.34 (1.79)	1.33 (1.97)	1.13 (1.70)	1.50 (2.19)	1.13 (1.46)
Mean (SD)					

**Baseline Risk Factor Levels, Mean (SD)** ^b^					
Systèm International (SI) Units					
Mean Weight, kg	95.0 (15.8)	94.3 (17.2)	95.1 (18.4)	93.6 (16.2)	95.6 (18.5)
Mean BMI, kg/m^2^	33.2 (5.6)	33.6 (6.1)	34.1 (7.1)	33.1 (4.9)	34.7 (8.5)
Hemoglobin A1c, mmol/mol	44.5 (11.5)	43.8 (12.6)	42.4 (9.8)	45.2 (14.8)	42.5 (6.7)
Systolic Blood Pressure, mmHg	131 (15)	135 (18)	137 (18)	133 (18)	135 (20)
Total Cholesterol, mmol/L	4.69 (1.15)	4.79 (1.17)	4.74 (1.06)	4.84 (1.27)	5.20 (1.18)
HDL Cholesterol, mmol/L	1.33 (0.36)	1.42 (0.35)	1.45 (0.40)	1.39 (0.31)	1.38 (0.30)
Non-HDL Cholesterol, mmol/L	3.36 (1.07)	3.37 (1.06)	3.29 (0.91)	3.45 (1.18)	3.82 (1.19)
-					
Conventional Units					
Mean Weight, lbs	209.0 (34.8)	207.5 (37.8)	209.2 (40.5)	205.9 (35.6)	210.4 (40.8)
Hemoglobin A1c, %	6.2 (1.1)	6.2 (1.2)	6.0 (0.9)	6.3 (1.4)	6.0 (0.6)
Total Cholesterol, mg/dL	181.3 (44.3)	185.4 (45.3)	183.2 (41.0)	187.3 (49.2)	201.1 (45.4)
HDL Cholesterol, mg/dL	51.3 (14.1)	54.9 (13.7)	56.1 (15.5)	53.8 (12.0)	53.4 (11.7)
Non-HDL Cholesterol, mg/dL	130.0 (41.4)	130.4 (40.9)	127.1 (35.2)	133.5 (45.6)	147.8 (45.9)

* Gender and race/ethnicity based upon self-reported values stored in each patient’s electronic chart; weight, blood pressure, hemoglobin A1c, and cholesterol values based upon last recorded value within the past 12 months in each patient’s electronic chart; BMI is based on the last recorded weight and height in each patient’s electronic chart

† 30 of the 80 trial participants were randomly selected and approached for interviews to recruit 15 respondents

‡ Charlson comorbidity score is a weighted score based on the number of comorbid diagnoses ^29^

b values are based on electronic health records; 100% of patients had a weight and systolic blood pressure value in the past 12 months; A1c values were available for 338, 67, 33, 34, & 13 and cholesterol values were available for 418, 78, 37, 41, and 14 patients in the eligible, enrolled, BLS, CLS, and interviewed groups, respectively

## Key Messages Regarding Feasibility

1. What uncertainties existed regarding the feasibility?
  - It was unclear if feasible to use existing primary care staff members and technology tools to support weight loss goal-setting, remote monitoring of self-weighing, and adaptive weight loss coaching in ways that would be acceptable, feasible, and sustainable from the perspectives of leaders, managers, and staff
  - It was unclear if it would be technically feasible for an electronic health record system to automatically receive and analyze remote weight monitoring data from electronic scales
  - It was unclear if under a IRB waiver of written informed consent, an electronic health record system could identify, enroll, and randomly assign eligible patients to receive different forms of weight management services and supports
  - It was unclear what proportion of adult primary care patients eligible for healthy weight loss services would have biometric and metabolic risk indicators captured by the electronic health record system in ways that would prove useful for a rigorous outcome data analysis
2. What are the key feasibility findings?
  - It is feasible to engage health care and fitness industry leaders, managers, and frontline personnel to develop implementation strategies for core components of intensive lifestyle interventions that are both aligned with the prior evidence-base and acceptable, appropriate, and sustainable from the perspectives of implementers.
  - It is feasible to utilize existing electronic health record tools, primary care personnel, and workflows to deliver and coordinate services and supports for weight loss goal setting, selfmonitoring, and adaptive coaching that are consistent with evidence-based core components of proven-effective intensive lifestyle interventions
  - It is feasible to use electronic health record systems to collect metabolic and biometric outcome measures useful for a pragmatic clinical trial
  - It is feasible to use an electronic health record system to “silently” identify, enroll, and randomize eligible patients to receive different “minimal risk” lifestyle intervention services
3. What are the implications of the feasibility findings for the design of the main study?
  - The use of an electronic health record system to organize, deliver, and coordinate both digital and low-technology practice components that align with core elements of proven-effective intensive lifestyle interventions warrants further study in a larger, longer-duration, randomized effectiveness trial
  - It is feasible to design larger, future trials to utilize pragmatic eligibility assessment, enrollment, randomization, and data acquisition
